# Climate projections of human thermal comfort for indoor workplaces

**DOI:** 10.1007/s10584-024-03685-7

**Published:** 2024-02-07

**Authors:** Markus Sulzer, Andreas Christen

**Affiliations:** Chair of Environmental Meteorology, Department of Earth and Environmental Sciences, Faculty of Environment and Natural Resources, University of Freiburg, 79085 Freiburg, Germany

**Keywords:** Indoor climate, Thermal comfort, Occupational safety, Climate change, Heat stress, Climate projections

## Abstract

Climate models predict meteorological variables for outdoor spaces. Nevertheless, most people work indoors and are affected by heat indoors. We present an approach to transfer climate projections from outdoors to climate projections of indoor air temperature (*T*_i_) and thermal comfort based on a combination of indoor sensors, artificial neural networks (ANNs), and 22 regional climate projections. Human thermal comfort and *T*_i_ measured by indoor sensors at 90 different workplaces in the Upper Rhine Valley were used as training data for ANN models predicting indoor conditions as a function of outdoor weather. Workplace-specific climate projections were modeled for the time period 2070–2099 and compared to the historical period 1970–1999 using the same ANNs, but ERA5-Land reanalysis data as input. It is shown that heat stress indoors will increase in intensity, frequency, and duration at almost all investigated workplaces. The rate of increase depends on building and room properties, the workplace purpose, and the representative concentration pathway (RCP2.6, RCP4.5, or RCP8.5). The projected increase of the mean air temperature in the summer (JJA) outdoors, by + 1.6 to + 5.1 K for the different RCPs, is higher than the increase in *T*_i_ at all 90 workplaces, which experience on average an increase of + 0.8 to + 2.5 K. The overall frequency of heat stress is higher at most workplaces than outdoors for the historical and the future period. The projected hours of indoor heat stress will increase on average by + 379 h, + 654 h, and + 1209 h under RCP2.6, RCP4.5, and RCP8.5, respectively.

## Introduction

Anthropogenic climate change will not only cause an increase in mean global near-surface air temperature (*T*_a_, °C), but also heat waves will occur more frequently in the future, with greater duration and intensity (Costello et al. 2009; Arnell et al. 2019; Hertig et al. 2023). Heat stress can negatively impact human health and productivity as well as the economy (Fisk and Rosenfeld 1997; Institute of Medicine 2011; Lundgren et al. 2013; Venugopal et al. 2016). Health issues caused by heat exposure include heat strokes, heat exhaustion, mental health issues, and the worsening of pre-existing health conditions (Kjellstrom et al. 2009b; Gubernot et al. 2014; McGregor et al. 2015; Hertig et al. 2023). High heat exposure combined with high work intensity can cause major health issues and even death (Kjellstrom et al. 2009a). Increased morbidity and mortality caused by heat is expected in the future (Costello et al. 2009).

Because people spend most of their occupational time indoors, it is important to take the indoor conditions into account when discussing human thermal comfort and heat stress under future conditions (Rosenfelder et al. 2016; Ackermann and Matzarakis 2021). Indoor human thermal comfort is influenced by multiple factors in addition to the outdoor meteorological conditions such as occupancy, orientation of the room, floor of the building, building-design, air conditioning, heating, volume, as well as the building’s surrounding area, including vegetation and building density (Institute of Medicine 2011; Barbosa et al. 2015; Sulzer et al. 2022; Leichtle et al. 2023). One of the major challenges for building designers in the future will be to design buildings which are both low-energy and can provide thermal comfort indoors (Holmes and Hacker 2007).

Because *T*_a_ is not the only meteorological variable affecting human thermal comfort, different thermal indices are used in biometeorological studies (Burton et al. 2009; Johansson et al. 2014; Staiger et al. 2019; Matzarakis 2021). A common thermal comfort index is the physiologically equivalent temperature (PET, °C), which is based on the Munich Energy Balance Model for Individuals (MEMI) and can be applied indoors and outdoors (Höppe 1999; Johansson et al. 2014; Matzarakis et al. 2016). Since the unit of PET is °C, the index is suited to communicate to employees and managers, especially for those who are not familiar with human-biometeorological terminology (Matzarakis et al. 1999; Matzarakis 2021). Along with personal variables (age, height, weight, gender, clothing, activity), the meteorological variables *T*_a_, relative humidity (*RH*, %), wind velocity (*v*, m s^−1^), and mean radiant temperature (*T*_mrt_, °C) are used as inputs for calculating PET (Höppe 1999).

To date, human biometeorological studies calculating thermal comfort indices for future climate projections generally consider outdoor conditions only (Matzarakis and Amelung 2008; Matzarakis and Endler 2010; Cheung and Hart 2014; Wallenberg et al. 2023; Bal and Kirchner 2023; Çağlak et al. 2023). Climate projections enable the modeling of PET changes in the future for certain locations (Matzarakis and Endler 2010) or worldwide (Matzarakis and Amelung 2008). For example, Wallenberg et al. (2023) used Coordinated Regional Downscaling Experiment data of the European Domain (EURO-CORDEX) to model thermal stress indices, including PET, at a shaded and a sunlit outdoor location in five Swedish cities to estimate the future heat stress of preschoolers.

However, thermal outdoor conditions are not representative for indoor thermal comfort. Further, different workplaces at indoor locations in the same area can also vastly differ from each other, due to various building and room properties or machines generating waste heat (Institute of Medicine 2011; Barbosa et al. 2015; Sulzer et al. 2022). A couple of studies assessed indoor air temperature (*T*_i_, °C) under future climates (Barbosa et al. 2015; Liu et al. 2020a, b; Muñoz González et al. 2020; Jafarpur and Berardi 2021; Lei et al. 2022). All studies modeling future indoor conditions to date were using building energy models (in most cases “EnergyPlus” (EnergyPlus 2023)), which requires, among other factors, detailed information about room and building geometry, wall material, and energy systems (Barbosa et al. 2015; Liu et al. 2020a, b; Muñoz González et al. 2020; Jafarpur and Berardi 2021; Lei et al. 2022; Liu et al. 2023).

We present an approach to project future indoor conditions without the need of a building energy model, but instead based on artificial neural network (ANN) models, trained with meteorological outdoor data and indoor data measured at different real workplaces, without using any additional information about the rooms and buildings.

In a previous study, we showed that low-cost indoor sensors can be used for nowcasting thermal comfort at different indoor workplaces (Sulzer et al. 2022) and as training data for ANN models to forecast short-term indoor thermal comfort and issue indoor heat warnings (Sulzer et al. 2023). The goal of this article is to expand the short-term forecasting approach of Sulzer et al. (2023) to calculate workplace-specific climate projections using low-cost sensors and ANN models. We present climate projections for 90 indoor workplaces located in the Upper Rhine Valley in Central Europe, which are classified into offices, laboratories and workshops, production, storage and logistics, and agriculture and forestry.

For all 90 workplaces, we compare statistics of *T*_i_ and indoor physiologically equivalent temperature (PET_i_) for the projected future (2070–2099, based on 22 different climate projections covering three representative concentration pathways (RCPs)), to a historical 30-year reference period (1970–1999) to answer the following questions:

- What is the expected increase in* T*_i_ and PET_i_ across different workplaces between the historical and projected future periods?
- Is the projected increase of *T*_i_ at the workplaces lower or higher than the increase of *T*_a_ outdoors?
- What is the variability of expected indoor climate changes between different workplaces with regard to *T*_i_ and PET_i_?
- How do exceedances of selected thresholds for *T*_i_ and PET_i_ change for different workplaces in the future?
- How does the frequency, duration, and intensity of heat indoors change depending on workplace context?

## Methodology

The ANN models used to generate the workplace-specific climate projections were built with the python package Keras using TensorFlow (Christin et al. 2019). The ANNs consisted of an input layer, a hidden layer, and an output layer. The single hidden layer used a sigmoid activation function, and the output layer a linear activation function. In the hidden layer were 16 neurons and a batch size of eight was used. The ANNs used the optimizer “adam” (Kingma and Ba 2017) with a default learning rate of 0.0001. The structure of the ANNs is the same as for the workplace-specific short-term indoor heat health warnings presented in Sulzer et al. (2023) where further details on the technical aspects of the ANNs and their evaluation can be found, including the testing of different input variables and a variable importance analysis.

The target data used for the training of the ANN models were measured by a custom-made low-cost sensor system, the “Mobile Biometeorological System” (MoBiMet), from Spring 2021 to Autumn 2022 in the Upper Rhine Valley region: at 51 workplaces in the area of Freiburg, Germany; 25 workplaces in the area of Basel, Switzerland; and 14 workplaces in the area of Strasbourg, France (Sulzer et al. 2022, 2023). The majority of the workplaces used for this study are offices (53), but also workplaces in laboratories and workshops (8), production (7), storage and logistics (10), as well as agriculture and forestry (12) were equipped with MoBiMets. The MoBiMets used calibrated low-cost sensors to measure *T*_i_, *RH*, globe temperature (*T*_g_, °C) to calculate *T*_mrt_, and at workplaces with a high level of ventilation, mainly in agriculture and forestry, also *v* (Sulzer et al. 2022). PET_i_ was calculated directly on the MoBiMets by the measurements of the sensors using the python code of Walther and Goestchel (2018) for a standardized person (Matzarakis et al. 2011). Sixty-nine of the 90 workplaces had active heating systems for the cold period, but only 3 of the 90 workplaces had manually operated active cooling systems.

For each workplace, two separate ANN models were trained, one for *T*_i_ and the other for PET_i_. The input data for the ANN models are hourly ERA5-Land data (Muñoz Sabater 2019) of the grid cells of Freiburg (48.0°N, 7.8°E), Basel (47.6°N, 7.6°E), and Strasbourg (48.6°N, 7.7°E). The meteorological variables used as input for the ANN models are *T*_a_ and dew point temperature (*T*_d_, °C) at 2 m above the surface, *v* at 10 m above the surface, and atmospheric pressure (*p*, hPa), global irradiance (*G*, W m^−2^), and long-wave downwelling radiation (*A*_G_, W m^−2^) at the surface. In addition to the meteorological data at target time *t*, the ANN models consider the data of *T*_a_, *G*, and *A*_G_ 3 h, 6 h, 12 h, and 24 h prior to *t*. The ANNs were trained to reproduce the available target data *T*_i_ and PET_i_ measured by the specific MoBiMet at each workplace for the period from February 2021 to October 2022 (in all cases, the MoBiMets were operated for at least one full year) by using the time-corresponding ERA5-Land data of the matching grid cell of Basel, Freiburg, or Strasbourg for each workplace.

Before building the ANN models for each workplace, the available data from the respective MoBiMet were randomly divided into training (80%), validation (10%), and test data sets (10%). During the training process, the weights are repeatedly adjusted to minimize the errors between the modeled output of the ANN and the target data provided in the training data. The validation data was used to counteract overfitting, by implementing an early stopping callback. The test data set is used in the end, after the training is completed, to compare the modeled data to the target data of a totally unknown data set and rate the ANN model’s accuracy (Chollet 2018). The mean absolute error (MAE) between the *T*_i_ modeled by the ANNs and the *T*_i_ measured by the sensors at the different workplaces in the independent test data set ranges between 0.39 and 2.76 K. The average MAE at all workplaces is 0.88 K. The MAE of the ANNs modeling PET_i_ ranges from 0.40 to 2.41 K and the average MAE of all workplaces is 0.96 K.

For future periods, EURO-CORDEX data are used for the input to the ANNs. EURO-CORDEX data are climate projections downscaled to a horizontal resolution of 0.11° (Giorgi and Gutowski 2015). In total, 22 EURO-CORDEX climate projections were selected, which were available at a temporal resolution of 3 h at the Copernicus climate data store (Copernicus Climate Change Service, Climate Data Store 2019). These include nine combinations of six different general circulation models (GCMs), three different regional climate models (RCMs), and two ensemble members (r1i1p1 and r12i1p1) for the available data of the three RCPs 2.6, 4.5, and 8.5 (Table 1).

**Table 1:** 3-h EURO-CORDEX data used as input for the ANNs to model future climate at different workplaces in the Upper Rhine Valley (Copernicus Climate Change Service, Climate Data Store 2019)

#	Global climate model	Regional climate model	Ensemble member	Experiments
1	CCCma-CanESM2	CLMcom-CCLM4-8–17	r1i1p1	RCP8.5
2	ICHEC-EC-EARTH	KNMI-RACMO22E	r1i1p1	RCP4.5, RCP8.5
3	ICHEC-EC-EARTH	KNMI-RACMO22E	r12i1p1	RCP2.6, RCP4.5, RCP8.5
4	ICHEC-EC-EARTH	SMHI-RCA4	r12i1p1	RCP2.6, RCP4.5, RCP8.5
5	IPSL-IPSL-CM5A-MR	SMHI-RCA4	r1i1p1	RCP4.5, RCP8.5
6	MIROC-MIROC5	CLMcom-CCLM4-8–17	r1i1p1	RCP2.6, RCP8.5
7	MOHC-HadGEM2-ES	KNMI-RACMO22E	r1i1p1	RCP2.6, RCP4.5, RCP8.5
8	MOHC-HadGEM2-ES	SMHI-RCA4	r1i1p1	RCP2.6, RCP4.5, RCP8.5
9	MPI-M-MPI-ESM-LR	SMHI-RCA4	r1i1p1	RCP2.6, RCP4.5, RCP8.5

**Fig. 1:**
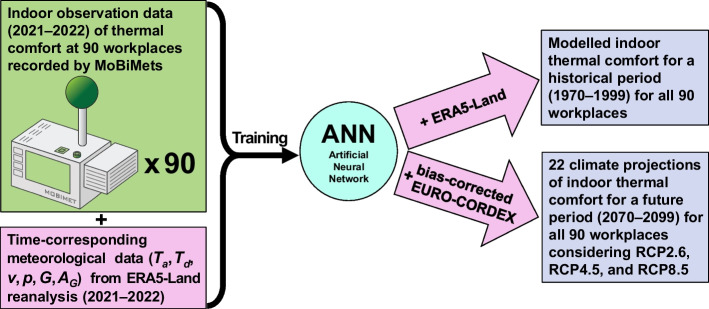
Methodology using sensor data, artificial neural networks, and gridded climate data for modeling indoor thermal comfort for historical and future periods at 90 workplaces

The climate projection data nearest to the used ERA5-Land grid cell of Freiburg, Basel, and Strasbourg were extracted from the EURO-CORDEX data. To make EURO-CORDEX data comparable to ERA5 data, EURO-CORDEX data were bias corrected relative to ERA5-Land data before their use as input data for the ANNs. ERA5-Land data of 1970–1999 at a temporal resolution of 3 h were used as input data for workplace-specific ANN models to calculate the indoor thermal comfort at every workplace for the entire 30-year historical period 1970–1999 and to adjust the EURO-CORDEX data to the ERA5-Land data according to the quantile delta mapping (QDM) method using the historical modeled EURO-CORDEX data from 1970 to 1999 for all used variables of each climate projection (Cannon et al. 2015; Fauzi et al. 2020; Qian and Chang 2021). QDM is an improved version of quantile mapping (Qian and Chang 2021; Tong et al. 2021), a commonly used method in climate downscaling to bias-correct climate projections against observations (Thrasher et al. 2012; Fiddes et al. 2022). The R package “MBC: Multivariate Bias Correction of Climate Model Outputs” was used to perform the QDM bias correction (Cannon 2020). Next, bias-corrected data for the period from 2070 to 2099 was used as input data for the ANN models to generate the workplace-specific indoor climate projections. Six RCP2.6, seven RCP4.5, and nine RCP8.5 climate projections of each workplace were used to project the future time period from 2070 to 2099. It is an inherent part of the proposed data-driven methodology that all projections shown assume no changes to the building, building technology, or building surrounding. The dataflow for modeling indoor thermal comfort at the 90 workplaces for the historical and future periods is summarized in Fig. 1.

For calculating the frequency of indoor heat exposure, different exceedance thresholds were defined for *T*_i_ and PET_i_. The used exceedance thresholds for *T*_i_ were ≥ 26 °C, ≥ 30 °C, and ≥ 35 °C, according to the German Federal Institute for Occupational Safety and Health (BAuA 2022). At *T*_i_ ≥ 26 °C, heat mitigation measures are recommended, and at *T*_i_ ≥ 30 °C, they are legally required (BAuA 2022). At *T*_i_ ≥ 35 °C, working in this room without technical or organizational measures or personal safety equipment is prohibited (BAuA 2022). The exceedance thresholds for PET_i_ were ≥ 23 °C, ≥ 29 °C, ≥ 35 °C, and ≥ 41 °C: the human physiological stress thresholds for slight, moderate, strong, and extreme heat stress, respectively, according to Matzarakis et al. (1999). Because the temporal resolution of the modeled data was 3 h, the values exceeding a threshold were multiplied by three to convert them into exceedance hours per year. For the data analysis, the data of all climate projections is normalized to a “noleap” calendar. When the daily maximum value of PET_i_ exceeds one of the thresholds, it was counted as a heat day with the respective category. This was used to investigate the change in total heat days per year and consecutive heat days per year for the time periods 1970–1999 and 2070–2099.

## Results

### Climate projections of air temperature at workplaces and outdoor air temperature

Table 2 contains the modeled mean values and ranges of average indoor air temperature (*T*_i_) at similar workplace types alongside the mean values and ranges of average outdoor air temperature (*T*_a_) of the three cities in which the workplaces are located. The data are averaged for the full annual cycle, winter months (DJF), and summer months (JJA) separately for the period 1970–1999, and all climate projections within a given RCP are further combined into RCP2.6 (*n* = 6), RCP4.5 (*n* = 7), and RCP8.5 (*n* = 9) ensembles for the period 2070–2099. The mean value of the modeled *T*_i_ averaged over all workplaces is expected to rise for all three RCPs compared to the historical period (1970–1999).

**Table 2:** Average values and ranges of the mean indoor air temperature (*T*_i_, °C) for workplaces of a similar type, modeled by the ANNs using ERA5-Land data as input data for 1970–1999 and the RCP2.6, RCP4.5, and RCP8.5 ensembles of EURO-CORDEX data for 2070–2099 considering all data (full year), winter (DJF), and summer (JJA) months. Mean values and ranges of the average outdoor air temperature (*T*_a_, °C) for Freiburg, Germany, Basel, Switzerland, and Strasbourg, France, are also shown

Model input data	Time of year	Production	Workshop and laboratory	Office	Storage and logistics	Agriculture and forestry	Outdoors
ERA5-Land (1970–1999)	Full year	24.6 (17.6–32.9)	22.0 (20.4–23.1)	22.5 (20.0–26.3)	17.9 (11.9–22.0)	13.3 (10.0–19.4)	9.6 (9.4–10.0)
	DJF	21.7 (10.7–32.0)	20.1 (17.3–22.7)	20.9 (17.3–25.8)	13.8 (4.0–19.1)	7.1 (2.7–16.2)	1.6 (1.2–2.1)
	JJA	27.9 (23.6–35.9)	24.4 (23.2–26.0)	24.7 (21.7–27.7)	22.5 (20.3–25.4)	20.3 (17.9–23.3)	18.0 (17.9–18.1)
RCP2.6 ensemble (2070–2099)	Full year	25.1 (18.6–33.3)	22.3 (20.9–23.3)	22.7 (20.1–26.4)	18.5 (13.1–22.5)	14.3 (11.0–22.0)	11.1 (10.9–11.4)
	DJF	22.1 (11.7–32.2)	20.3 (17.6–22.6)	21.0 (17.5–25.6)	14.3 (5.1–19.4)	7.9 (3.8–16.5)	3.2 (3.0–3.6)
	JJA	28.9 (24.6–36.1)	25.1 (24.0–26.7)	25.4 (21.3–28.2)	23.5 (21.7–26.2)	21.6 (19.2–24.4)	19.6 (19.4–19.7)
RCP4.5 ensemble (2070–2099)	Full year	25.5 (19.3–33.7)	22.6 (21.2–23.5)	23.0 (20.2–26.5)	19.0 (14.0–22.8)	15.0 (12.0–20.4)	12.1 (11.9–12.3)
	DJF	22.5 (12–5–32.2)	20.5 (17.9–22.6)	21.1 (17.7–25.6)	14.7 (6.1–19.7)	8.6 (4.8–16.7)	4.5 (4.4–4.8)
	JJA	29.3 (25.2–36.9)	25.5 (24.4–27.1)	25.8 (21.4–28.4)	23.9 (22.0–26.6)	22.2 (19.9–25.0)	20.3 (20.1–20.4)
RCP8.5 ensemble (2070–2099)	Full year	26.4 (20.7–34.8)	23.3 (22.0–24.1)	23.6 (20.4–26.9)	19.9 (15.8–23.5)	16.5 (13.7–21.4)	14.0 (13.8–14.3)
	DJF	22.9 (13.5–32.4)	20.7 (18.3–22.5)	21.2 (18.0–25.5)	15.3 (7.4–20.0)	9.6 (6.2–17.1)	6.0 (5.8–6.3)
	JJA	30.9 (27.0–39.6)	26.8 (25.6–28.4)	26.8 (21.4–30.1)	25.3 (22.5–27.7)	24.4 (22.1–27.1)	23.1 (22.9–23.3)

The modeled rise until the future period (2070–2099) compared to the historical period, averaged over all workplaces, is + 0.5 K, + 0.8 K, and + 1.6 K for the RCP2.6, RCP4.5, and RCP8.5 ensembles, respectively. The projected increase is lower in winter and higher in summer. The expected rise between 1970–1999 and 2070–2099 of mean *T*_i_ averaged over all workplaces is + 0.3 K, + 0.5 K, and + 0.9 K during the winter months and + 0.8 K, + 1.2 K, and + 2.5 K during the summer months for the RCP2.6, RCP4.5, and RCP8.5 ensembles, respectively. In contrast, the projected mean values of the full annual cycle of outdoor *T*_a_ are projected to increase by + 1.5 K, + 2.4 K, and + 4.3 K in Freiburg; + 1.5 K, + 2.5 K, and + 4.5 K in Basel; and + 1.4 K, + 2.3 K, and + 4.3 K in Strasbourg for the RCP2.6, RCP4.5, and RCP8.5 ensembles, respectively. Thus, for the full year, the projected annual outdoor *T*_a_ is increasing more than *T*_i_ averaged over all workplaces. For the RCP2.6 and RCP4.5 ensembles, the projected increase of mean *T*_a_ is higher during the winter compared to the summer. In contrast, for the RCP8.5 ensemble, the projected increase of outdoor *T*_a_ during the summer months is up to + 1.0 K higher than during winter, which corresponds to a projected increase of mean *T*_a_ during the summer month of + 5.1 to + 5.4 K in the three cities. The differences between the three cities’ projected *T*_a_ increases are small, given their similar location in the Upper Rhine Valley within ~ 120 km and elevation between 140 and 280 m above sea level. The projected increase in *T*_i_ at the workplaces in agriculture and forestry is closest to the projected increase in *T*_a_. The workplaces in agriculture and forestry are also the type of workplaces with the highest mean *T*_i_ increase, rising by + 3.2 K on average considering the most extreme RCP8.5 ensemble. The lowest increase for the RCP8.5 ensemble is projected in offices, by an average of + 1.1 K. Offices are also the type of workplaces with the lowest *T*_i_ increase for the summer, by + 2.1 K, and winter months, by + 0.3 K on average considering the RCP8.5 ensemble.

In Table 3, the average number and range of mean exceedance hours per year (hours in which *T*_i_ exceeds the thresholds of ≥ 26 °C, ≥ 30 °C, and ≥ 35 °C) are shown for the different workplace types. This is compared to the number of hours per year in which *T*_a_ exceeds the same thresholds outdoors.

**Table 3:** Mean and range of the average number of hours per year exceeding *T*_i_ of 26 °C, 30 °C, and 35 °C at workplaces of a similar type, modeled by the ANNs using ERA5-Land data for 1970–1999 and the RCP2.6, RCP4.5, and RCP8.5 ensembles of EURO-CORDEX as input data for 2070–2099. Also, mean and range of the average number of hours per year exceeding the *T*_a_ thresholds per year are shown for the outdoor situation in Freiburg, Germany, Basel, Switzerland, and Strasbourg, France

Model input data	*T*_i_ threshold	Production	Workshop and laboratory	Office	Storage and logistics	Agriculture and forestry	Outdoors
ERA5-Land (1970–1999)	≥ 26 °C	3673 (544–8740)	594 (176–1273)	813 (0–4513)	348 (0–952)	403 (184–817)	141 (132–148)
	≥ 30 °C	2265 (7–7669)	30 (0–101)	74 (0–474)	42 (0–249)	107 (24–309)	17 (15–19)
	≥ 35 °C	526 (0–2297)	0 (0–0)	2 (0–57)	1 (0–11)	7 (0–44)	0 (0–0)
RCP2.6 ensemble (2070–2099)	≥ 26 °C	3891 (846–8741)	918 (405–1668)	1120 (0–4408)	547 (0–1379)	534 (262–1034)	273 (262–281)
	≥ 30 °C	2415 (54–7743)	94 (0–303)	143 (0–692)	90 (0–420)	164 (44–403)	62 (59–65)
	≥ 35 °C	617 (0–2380)	0 (0–0)	4 (0–91)	4 (0–35)	14 (0–73)	4 (3–6)
RCP4.5 ensemble (2070–2099)	≥ 26 °C	4066 (1068–8749)	1139 (520–1975)	1337 (0–4546)	676 (0–1661)	670 (354–1228)	372 (354–388)
	≥ 30 °C	2537 (79–7800)	130 (0–392)	181 (0–835)	115 (0–485)	222 (63–525)	95 (89–98)
	≥ 35 °C	707 (0–2583)	0 (0–0)	5 (0–120)	5 (0–39)	21 (0–99)	7 (6–10)
RCP8.5 ensemble (2070–2099)	≥ 26 °C	4504 (1729–8752)	1833 (1115–2712)	1966 (0–4822)	1140 (0–2379)	1077 (641–1734)	785 (760–818)
	≥ 30 °C	2869 (204–7984)	356 (0–872)	359 (0–1375)	262 (0–892	450 (175–882)	313 (300–332)
	≥ 35 °C	929 (0–3001)	0 (0–0)	11 (0–284)	17 (0–94)	64 (0–249)	61 (59–64)

The highest number of heat stress hours per year can, on average, be found at workplaces of the type production for all three *T*_i_ thresholds and all the different model input data used. The average lowest number of hours per year above 26 °C in the modeled historical time period can be found at the workplaces of type storage and logistics. For all three future RCPs, the lowest number of hours per year above 26 °C can be expected at the workplaces in agriculture and forestry. Values above the 30 °C exceedance threshold are least common in workshops and laboratories for the modeled years 1970–1999. In the modeled future ensembles of all three RCPs, *T*_i_ values ≥ 30 °C are least likely to appear at workplaces in storage and logistics, on average. In the modeled data, the average hours per year beyond 35 °C, in the historical and future period, are 0 h at all the workplaces in workshops and laboratories.

**Fig. 2:**
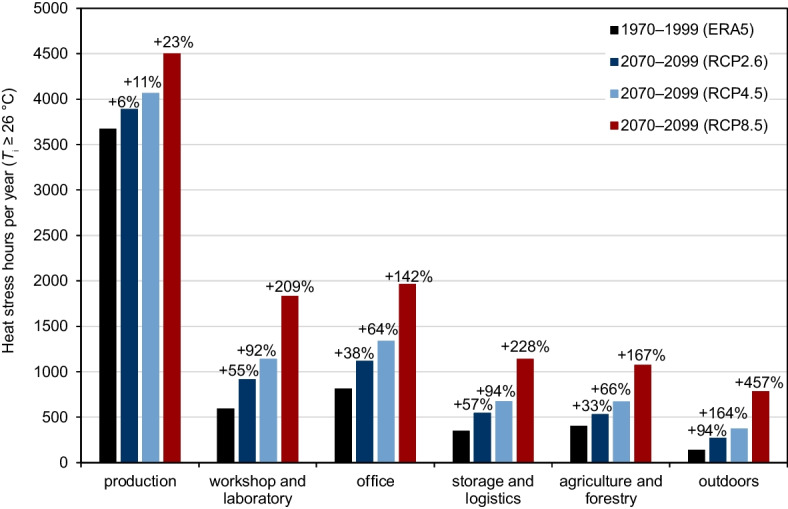
Mean heat stress hours per year exceeding *T*_i_ of ≥ 26 °C averaged for the different workplace types modeled by the ANNs using ERA5-Land data for 1970–1999 and the RCP2.6, RCP4.5, and RCP8.5 ensembles based on 22 EURO-CORDEX climate projections as input data for 2070–2099 as well as *T*_a_ ≥ 26 °C outdoors

Averaged over all workplaces the mean hours per year exceeding the value of 26 °C for *T*_i_ are rising for the modeled future time period, considering the RCP2.6, RCP4.5, and RCP8.5 ensembles, compared to the historical time period, by + 266 h (+ 29%), + 460 h (+ 51%), and + 1032 h (+ 114%), respectively. The projected mean numbers of hours per year averaged for the different workplace types exceeding the value of 26 °C for *T*_i_ in the historical and future time period, as well as the projected increase in percent, are shown in Fig. 2.

The projected increase averaged over all workplaces in hours per year with *T*_i_ values above 30 °C are + 71 h, + 117 h, and + 314 h for the RCP2.6, RCP4.5, and RCP8.5 ensembles, respectively. For the ≥ 35 °C *T*_i_ exceedance threshold, the projected increase in hours per year is + 10 h, + 18 h, and + 46 h for the different RCPs. Three workplaces show no *T*_i_ values above 26 °C in the modeled data for the historical and the future time periods; two of them are workplaces in offices equipped with active cooling systems. At 17 workplaces, *T*_i_ never exceeds 30 °C, and at 61 workplaces, *T*_i_ is also always under 35 °C in the modeled historical and future period. Outdoors *T*_a_ hours per year exceeding the ≥ 26 °C threshold rise by more than + 600 h, above 30 °C rise from < 20 to ≥ 300 h, and the 35 °C exceedance threshold rise from 0 h to an average of 61 h for the three cities considering the RCP8.5 ensemble. On average over all workplaces, the increase of heat stress hours per year exceeding the *T*_i_ thresholds is higher than for *T*_a_ outdoors, except for hours per year above 35 °C modeled by using the RCP8.5 ensemble.

### Workplace-specific climate projections of indoor physiologically equivalent temperature

#### Frequency and intensity of heat stress

**Fig. 3:**
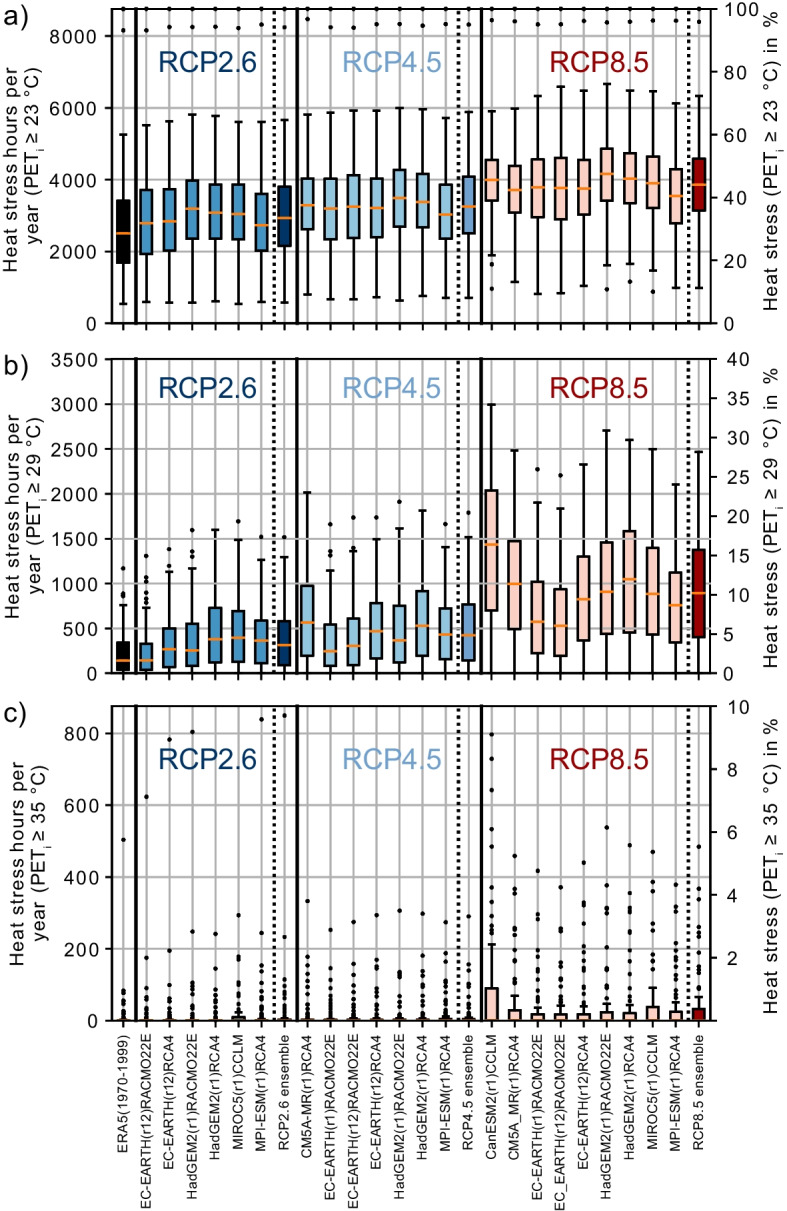
Boxplots showing the frequency of heat stress above the indoor physiologically equivalent temperature (PET_i_) exceedance thresholds of **a** ≥ 23 °C, **b** ≥ 29 °C, and **c** ≥ 35 °C at all modeled workplaces in hours per year (left axis) and in % (right axis), using the ERA5-Land data as input data for the time period from 1970 to 1999 and 22 EURO-CORDEX climate projections to model the time period from 2070 to 2099. All projections available per RCP are further shown as ensemble distributions for each RCP. Outliers in Fig. 3a showing hours per year for PET_i_ ≥ 23 °C above 8000 h represent two workplaces in production, which are exposed to waste heat from heavy machinery. Most outliers of the respective workplaces are not shown in Fig. 3b and c

Further in this article, we will present the results of the modeled PET_i_ at the workplaces to assess changes in thermal comfort. Averaged over all workplaces, the projected PET_i_ is expected to rise from 20.7 °C by + 0.4 K, + 0.7 K, and + 1.2 K for RCP2.6, RCP4.5, and RCP8.5 ensembles, respectively. In Fig. 3, the data of mean heat stress hours per year at all workplaces are summarized for the period 1970–1999 and the climate projections for the different RCPs and each individual ensemble member from 2070 to 2099 for PET_i_ ≥ 23 °C (Fig. 3a), PET_i_ ≥ 29 °C (Fig. 3b), and PET_i_ ≥ 35 °C (Fig. 3c).

The median number of exceedance hours per year for the threshold PET_i_ ≥ 23 °C is 2502 h, and the mean is 2662 h over all workplaces in the historical period. The median number of hours per year of all workplaces with PET_i_ ≥ 23 °C increases in the future time period under all ensembles by + 424 h (2736–3187 h), + 752 h (3033–3484 h), and + 1358 h (3541–4163 h) for RCP2.6, RCP4.5, and RCP8.5, respectively. For all workplaces, the median number of hours per year exceeding PET_i_ ≥ 29 °C modeled for the historical period 1970–1999 is 140 h. The median of hours per year exceeding PET_i_ ≥ 29 °C increases in all climate projections during the future period by + 174 h (145–365 h), + 285 h (243–565 h), and + 753 h (530–1435 h) under RCP2.6, RCP4.5, and RCP8.5 ensembles, respectively. The median of the number of heat hours per year for PET_i_ ≥ 35 °C for all modeled workplaces is 0 h; the mean is 34 h. The projected increase of the mean hours per year averaged over all workplaces is + 55 h, considering the most extreme RCP8.5 ensemble for 2070–2099.

**Fig. 4:**
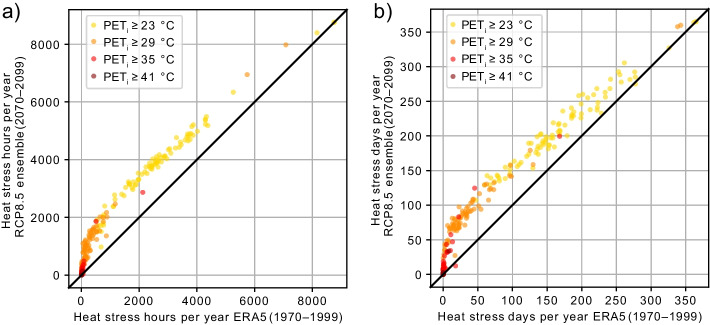
Scatter plot showing heat stress **a** hours and **b** days per year for the different PET_i_ exceedance thresholds at each workplace for the modeled time period from 1970 to 1999 using ERA5-Land data as model input and from 2070 to 2099 using the RCP8.5 ensemble as model input. Heat stress hours and days per year for the RCP8.5 ensemble are averaged over all nine selected EURO-CORDEX climate projections considering RCP8.5

Figure 4 compares the number of heat stress hours per year (Fig. 4a) and days per year (Fig. 4b) above the different PET_i_ exceedance thresholds, modeled by using the ERA5-Land data for 1970–1999 vs. the RCP8.5 ensemble for 2070–2099. A day was counted as a heat stress day when the daily maximum PET_i_ exceeded the corresponding PET_i_ threshold.

Heat stress hours per year above the PET_i_ ≥ 23 °C threshold occur at all workplaces modeled for 1970–1999. The projected increase averaged over all workplaces for PET_i_ ≥ 23 °C are + 1209 h, + 654 h, and + 379 h using the RCP8.5, RCP4.5, and RCP2.6 ensembles, respectively. In the median over all workplaces, the relative increase in hours with PET_i_ ≥ 23 °C from 1970–1999 to 2070–2099 is + 17%, + 30%, and + 54% using the RCP8.5, RCP4.5, and RCP2.6 ensembles. For RCP8.5 as the most extreme projection, the heat stress days per year for the exceedance threshold PET_i_ ≥ 23 °C will rise at 87 of 90 workplaces in the projected future. The increase of heat stress days per year above the PET_i_ ≥ 23 °C threshold is + 41.8 days using the RCP8.5 ensemble.

For the modeled historical data (1970–1999), 78 of the 90 workplaces show at least 1 h per year with an exceedance of PET_i_ ≥ 29 °C. The number of workplaces with at least 1 h per year of exceedance PET_i_ ≥ 29 °C increases to 84 for the projected data of 2070–2099 using the RCP8.5 ensemble as input. Averaged over all workplaces, the heat stress hours per year for PET_i_ ≥ 29 °C increase on average by + 668 h and days per year by + 44 days. For the exceedance threshold of PET_i_ ≥ 29 °C, neither the heat stress days nor hours per year decline at any of the workplaces using the RCP8.5 ensemble.

While PET_i_ values above 35 °C were expected only at 23 of the 90 workplaces for the period 1970–1999, 29 of all monitored workplaces have at least once a year a condition with PET_i_ values above 35 °C in the period 2070–2099, for RCP2.6 and RCP4.5 ensembles as well as 34 of the 90 workplaces for the RCP8.5 ensemble. Only two modeled workplaces exceeded heat hours per year of PET_i_ ≥ 41 °C for more than 1 h in the historical period, but two more will reach the limit PET_i_ ≥ 41 °C in the projected future period.

#### Duration of heat stress

In Table 4, the average and range of the different workplace types mean duration of consecutive heat days per year, using the same PET_i_ exceedance thresholds as before, are shown for the historical data (1970–1999) and the future (2070–2099) under RCP8.5. Averaged over all workplaces, the length of mean consecutive heat days per year is increasing by + 3.8 days for PET_i_ ≥ 23 °C, + 4.5 days for PET_i_ ≥ 29 °C, and + 0.5 days for PET_i_ ≥ 35 °C in the modeled future.

**Table 4:** Mean values and range of the number of consecutive days per year with daily maximum PET_i_ values exceeding 23 °C, 29 °C, and 35 °C, averaged over all workplaces for a given workplace type for the historical data (1970–1999) and for the future (2070–2099) considering the RCP8.5 ensemble

Model input data	PET_i_ threshold	Production	Workshop and laboratory	Office	Storage and logistics	Agriculture and forestry
ERA5-Land (1970–1999)	≥ 23 °C	129.2 (7.7–364.9)	11.7 (5.5–18.0)	11.8 (2.9–19.0)	9.1 (5.1–21.5)	5.3 (3.4–9.2)
	≥ 29 °C	12.1 (2.8–38.0)	3.0 (1.0–5.3)	2.9 (0.0–6.1)	2.1 (0.0–5.1)	3.0 (2.1–4.1)
	≥ 35 °C	2.7 (0.0–8.6)	0.0 (0.0–0.0)	0.3 (0.0–2.9)	0.5 (0.02.0)	1.6 (1.0–2.2)
RCP8.5 ensemble (2070–2099)	≥ 23 °C	140.0 (12.6–365.0)	14.4 (6.9–17.9)	14.1 (3.2–19.1)	14.9 (11.7–19.5)	10.4 (6.9–12.9)
	≥ 29 °C	31.3 (5.9–118.5)	6.9 (1.4–12.8)	6.2 (0.0–11.8)	4.7 (0.0–12.8)	5.7 (4.1–8.5)
	≥ 35 °C	4.3 (0.0–10.0)	0.6 (0.0–1.9)	0.4 (0.0–6.1)	1.1 (0.0–3.6)	2.5 (1.2–4.1)

#### Hourly variability

**Fig. 5:**
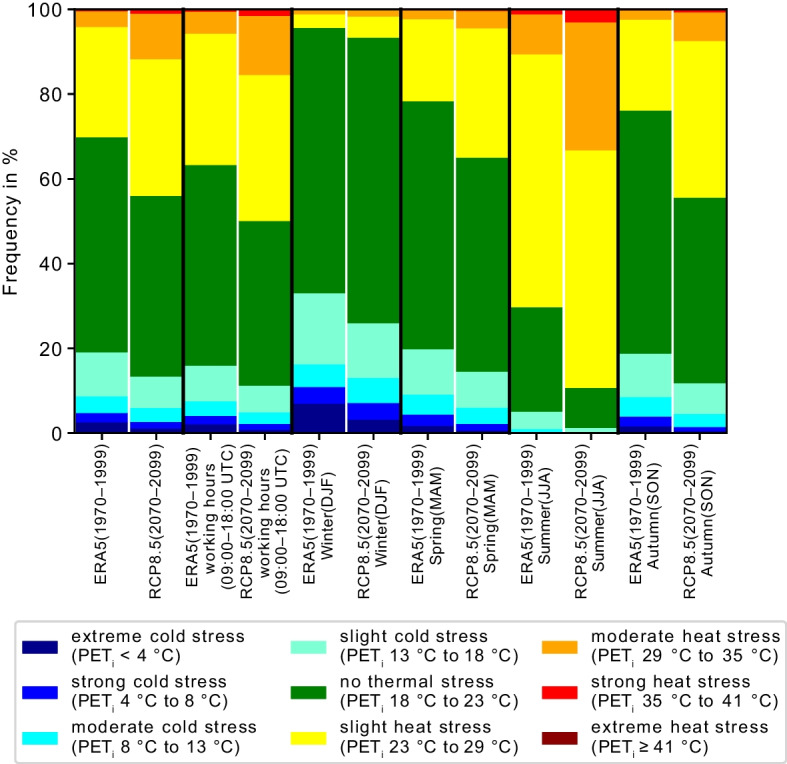
Frequency distributions of thermal stress levels according to PET_i_ over all workplaces for two time periods: 1970–1999 and 2070–2099. The frequency distribution of the future period shows the averaged frequency over all nine climate projections considering RCP8.5. The full years are compared to individual seasons as well as typical working hours (09:00–18:00 UTC)

Figure 5 shows the mean frequency of thermal comfort levels according to the PET_i_, averaged over all workplaces for 1970–1999 and for 2070–2099 using the RCP8.5 ensemble. Included are the full annual cycle, the different seasons winter (DJF), spring (MAM), summer (JJA), and autumn (SON), as well as typical working hours, defined as 09:00 UTC to 18:00 UTC.

Averaged over all workplaces, the proportion of hours with no thermal stress is 51% in the historical period 1970–1999, and this declines to 43%, 47%, and 49% in the projected future period (2070–2099) under RCP8.5, RCP4.5, and RCP2.6, respectively. On average, heat stress (PET_i_ ≥ 23 °C) occurs during 30% of the time in the historical period and increases to 44%, 38% and 35% in the projected future period for RCP8.5, RCP4.5, and RCP2.6, respectively. Only considering working hours (09:00–18:00 UTC), the portion without any thermal stress is 4% lower than for the full data set in the modeled historical and future periods. The fraction of heat stress during working hours is 37% for the years 1970–1999 and 50%, 44%, and 41% for 2070–2099 under RCP8.5, RCP4.5, and RCP2.6, respectively. Averaged over all workplaces, the portion of moderate heat stress or higher is expected to triple under RCP8.5, from 4 to 12%, and to 7% and 6% for RCP4.5 and RCP2.6 ensembles. During typical working hours, the occurrence of moderate heat stress or higher will increase from 6% in the modeled historical period to 15%, 10%, and 8% in the projected future period for RCP8.5, RCP4.5, and RCP2.6, respectively.

#### Seasonal variability

In all four seasons, heat stress (PET_i_ ≥ 23 °C) will be more frequent in the projected future period (2070–2099) compared to the historical period (1970–1999). In winter, the thermally comfortable portion (18 °C < PET_i_ < 23 °C) will rise from 63% in the historical period by + 4%, + 3%, and + 2% in the projected future period under RCP8.5, RCP4.5, and RCP2.6. Further, in winter, the increase in heat stress frequency is + 3% in the future period under RCP8.5 and + 1% under RCP4.5 and RCP2.6 compared to the historical period. This increase is less compared to other seasons. In spring, the frequency of heat stress averaged over all workplaces increases from 22% in the historical period to 35%, 29%, and 26% in the projected future period under RCP8.5, RCP4.5, and RCP2.6, respectively. In the modeled historical and projected future period, summer is most critical for heat stress. In the historical time period, on average, 70% of the summertime is characterized by heat stress, including 11% of moderate heat stress or higher (PET_i_ ≥ 29 °C). For the period 2070–2099, under RCP8.5, the average fraction of time projected to experience heat stress is 89% with 33% of moderate heat stress or higher. Similarly, for RCP4.5 and RCP2.6, the fraction of time projected to experience heat stress is 82% and 78%, with 20% and 16% of moderate heat stress or higher. The portion without thermal stress in summer declines from 25 to 9%, 16%, and 19% for RCP8.5, RCP4.5, and RCP2.6. The strongest projected relative increase of heat stress occurrence, by + 21% to a value of 44% for RCP8.5 and to 29%, and 24% for RCP4.5 and RCP2.6 is found in autumn. Also, time periods of moderate heat stress or higher rise from 2 to 7%, 4% and 3% in autumn under future projections for RCP8.5, RCP4.5, and RCP2.6. Simultaneously thermally comfortable conditions decrease from 57 to 44%, 51%, and 54% for the months in autumn under RCP8.5, RCP4.5, and RCP2.6.

#### Differentiation by workplace

**Fig. 6:**
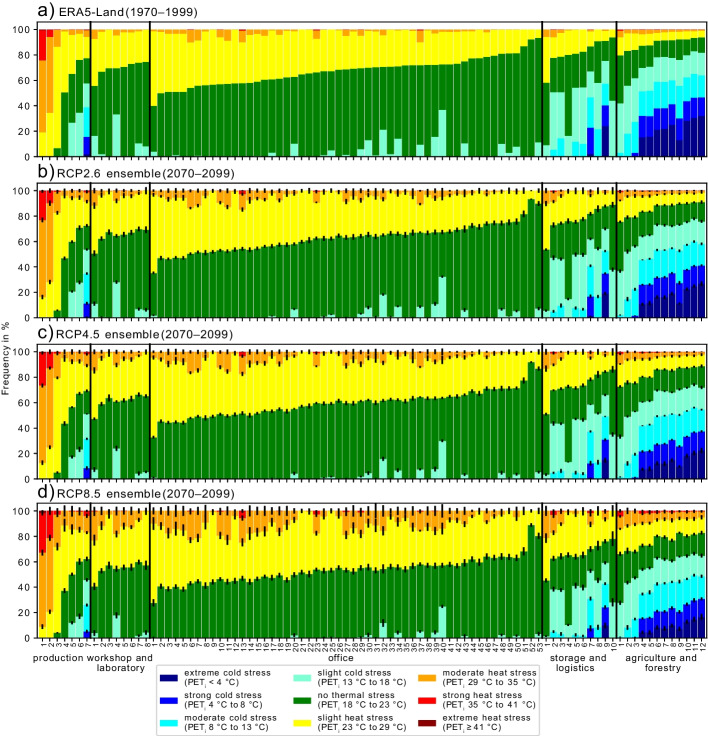
Frequency distributions of human thermal stress levels modeled by using **a** ERA5-Land data for 1970–1999 and ensemble distributions of all selected EURO-CORDEX climate projections considering **b** RCP2.6, **c** RCP4.5, and **d** RCP8.5 for 2070–2099 at every workplace. The black lines show the standard deviations of the individual ensemble members in a given RCP

The frequency of thermal stress levels according to PET_i_ is presented for each workplace separately in Fig. 6 for the historical data (Fig. 6a) and the RCP2.6 (Fig. 6b), RCP4.5 (Fig. 6c), and RCP8.5 (Fig. 6d) ensembles. The workplaces in Fig. 6 are organized by workplace type and, within the same type, by descending heat stress frequency (PET_i_ ≥ 23 °C) in the historical time period, from left to right. Figure 6 highlights the differences in thermal behavior at the 90 workplaces, during historical and future climate conditions, as well as the differences in shifts of thermal comfort classes under a changing climate. All workplaces have one aspect in common: heat stress will occur more frequently in the projected future, regardless of the considered RCP and type of workplace. But the frequency and intensity of the additional heat stress in the future show large variations between different workplaces, even within the same workplace type. Also, uncertainties increase for climate projections of higher RCPs, but this can also be an effect of the different numbers of climate projections available for the three RCPs.

**Fig. 7:**
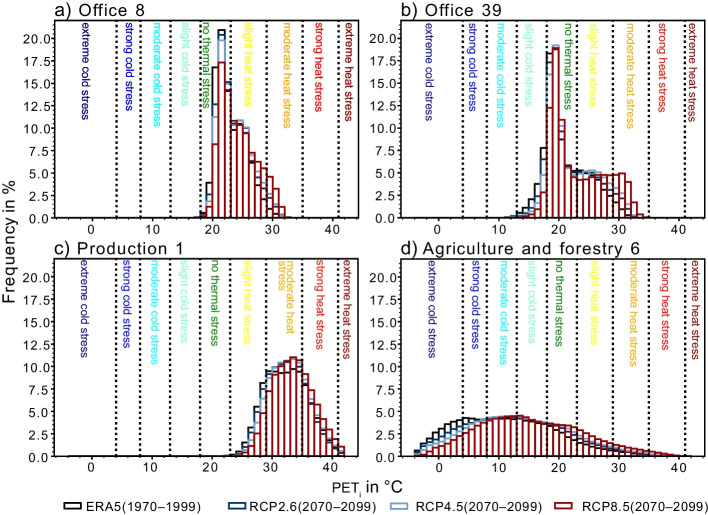
Frequency distributions of modeled PET_i_ classes with a bin size of 1 K at example workplaces in **a** an office on the ground floor, **b** an office on the second floor under a sloping roof, **c** production next to heavy machinery, and **d** agriculture in a cowshed, using ERA5-Land data for 1970–1999 and the RCP2.6, RCP4.5, and RCP8.5 ensembles for 2070–2099 as model input. The frequency distributions of the future period show averaged frequencies over all 6, 7, and 9 climate projections considering RCP2.6, RCP4.5, and RCP8.5, respectively

To further explore thermal comfort situations and projected changes expected in the future at different workplaces in more detail, examples of PET_i_ distributions are shown for four workplaces located in Freiburg, in Fig. 7. The distributions of the modeled 30-year historical (1970–1999) and future time periods (2070–2099) are presented for the RCP8.5, RCP4.5, and RCP2.6 ensembles. The example workplaces are located in an office on the first/ground floor (Fig. 7a), an office on the third/top floor directly under a sloping roof in the same building (Fig. 7b), a workplace in production (Fig. 7c), and a workplace in agriculture (Fig. 7d).

The building, in which the two presented offices are located in, was built around 1900 in Freiburg’s city center, mainly of stone. Both offices are single-occupancy and have a computer and a heating system. The office on the ground floor has windows facing west; the office on the third (top) floor has windows facing east. The all-year mean PET_i_ in the office on the ground floor is 23.1 °C and 21.0 °C on the top floor for the historical 1970–1999 period. Heat stress is more common on the ground floor, but mostly light heat stress. Cold stress and moderate heat stress or higher are more likely to appear in the office on the third floor. The projected rise of heat stress hours per year is similar in both offices: on the ground floor + 1332 h (+ 15.2%), + 729 h (+ 8.3%), and + 456 h (+ 5.2%); on top floor + 1349 h (+ 15.4%), + 771 h (+ 8.8%), + 420 h (+ 4.8%) for RCP8.5, RCP4.5, and RCP2.6, respectively. On the top floor, however, the projected increase in the occurrence of moderate heat stress or a higher stress level class, taking the RCP8.5 ensemble into account, is considerably higher with + 1130 h (+ 12.9%) compared to the projected increase of + 675 h (+ 7.7%) on the ground floor. Also, the projected mean PET_i_ rise of + 2.2 K is higher on the third floor than the projected rise of + 1.3 K on the ground floor, considering the RCP8.5 ensemble. The frequency of comfortable thermal conditions will decline in the projected future in both offices, even though the frequency of cold stress declines by up to − 12% in the office on the top floor.

The workplace in production is located on the third floor in a brick building built circa 1940 in a chemical factory complex in Freiburg’s industrial area. The workplace is next to heavy machinery generating waste heat and north-facing windows. The mean value of PET_i_ will increase from 32.4 °C in the historical period by + 0.1 K, + 0.5 K, and + 1.1 K in the projected future period, for the RCP2.6, RCP4.5, and RCP8.5 ensembles, respectively. The frequency of overall heat stress cannot increase at this workplace, because it is already at 100% for the modeled historical period and will stay at 100% for the modeled future. But the frequency of strong and extreme heat stress is expected to increase by + 8% and + 2%, respectively, when the data of the RCP8.5 ensemble are considered.

The workplace in agriculture is located approximately 10 km northeast of Freiburg. This is a workplace located in a cowshed built around 2000 with the main construction materials wood and concrete. The workplace is exposed to direct solar radiation from the west. Additional heat sources at this workplace are cows and occasionally a tractor. The PET_i_ values show a wide range of − 4.3 to 39.4 °C in the historical period, and a mean of 11.4 °C. In the future, the projected mean value will increase by + 1.3 K, + 2.3 K, and + 4.1 K for the RCP2.6, RCP4.5, and RCP8.5 ensembles. PET_i_ values in the comfortable range will become more common in the projected future, because of the decline in frequency of cold stress. But the frequency of heat stress at all levels is projected to increase under all RCPs.

## Discussion

All of the 90 workplaces exhibit a modeled increase of *T*_i_ that is less than the increase in *T*_a_ outdoors in all RCPs, especially during the winter months. This is obviously an effect of the space heating (in 69 of the 90 workplaces) but also partially the thermal inertia of buildings. The heat storage coefficients of the construction materials as well as shading of incoming solar radiation, among many other variables, can influence indoor thermal conditions. Nevertheless, averaged over all workplaces, the mean projected increase of *T*_i_ will be + 1.8 K, + 1.0 K, and + 0.5 K, for the RCP8.5, RCP4.5, and RCP2.6 ensembles, respectively. This generally leads to increased frequency of heat stress in the projected future at 89 of the 90 workplaces under RCP2.6 and at all workplaces under RCP4.5 and RCP8.5. Similar to the behavior of heat waves outdoors, which are expected to become more intense, more frequent, and longer in the future (Costello et al. 2009; Arnell et al. 2019; Hertig et al. 2023), heat stress indoors averaged over all workplaces will also be (a) more intense as mean values of *T*_i_ and PET_i_ are higher and shift to higher stress levels; (b) more frequent as shown by the projected increase in heat hours and days per year; and (c) longer, as the projected rise in duration of consecutive heat days per year implies.

The range of the projected increase of *T*_i_ for the different workplaces and RCPs is + 0.0 to + 3.9 K. The range of the projected mean PET_i_ increase is even wider, from + 0.1 to + 4.6 K. It can be confirmed that also in the modeled future, outdoor thermal conditions are not representative of indoor thermal conditions (Barbosa et al. 2015; Sulzer et al. 2022). Furthermore, different workplaces at indoor locations in the same area, even inside the same building, can differ from each other due to various building and room properties, for example floor of the building (Fig. 7a and b), orientation, occupancy, or waste heat (Barbosa et al. 2015; Sulzer et al. 2022). The wide range of workplace-specific climate projections imply that the outdoor conditions alone are not representative for indoors and the connection between outdoor and indoor conditions is specific for every indoor situation. The complex thermal behavior of indoor locations highlights the need for individual, context-specific climate projections for indoors, or at least many archetypes, to gain knowledge about the heat exposure of humans in the future. Figures 6 and 7 show that the changes of PET_i_ values in the future are complex: for example, not only can the median of the PET_i_ distribution shift in the projected future, but also its skewness (towards higher heat stress categories) compared to the data modeled for the historical period.

In most previous studies that attempted to project *T*_i_ and/or indoor thermal comfort changes for future periods, building energy models have been used. Building energy models are computationally expensive and require very detailed and some difficult to acquire information, which can lead to uncertainties, on/about the building’s geometry, construction materials, usage, energy systems, thermal conductivity, and occupancy to perform simulations (Barbosa et al. 2015; Liu et al. 2020a, b; Muñoz González et al. 2020; Jafarpur and Berardi 2021; Lei et al. 2022; Lin et al. 2023). Our data-driven approach to produce indoor climate projections based on ANNs trained on localized sensor data has the advantage that once trained, we could rapidly compute future thermal conditions at 90 workplaces with a resolution of 3 h over 30 years using 22 different climate projections. Our data-driven approach does not require any information about the room or building, not simplifying climate with typical meteorological years, just a single measuring device providing training data measured inside the specific room (for at least 1 year). The disadvantage of this approach is that properties of the room, occupancy, technology, or building envelope cannot be changed to test different adaption measures, as for example in the study by Barbosa et al. (2015), or the influence of the future climatic uncertainties on the efficiency of different adaption measures investigated (e.g., Liu et al. 2023). Nevertheless, a way of considering selected adaption measures such as improved shading, ventilation, or clothing for future PET_i_ modeling could be implemented by generating different ANN models for the four meteorological indoor variables needed to calculate PET_i_. Then, the input variables could be manipulated or modified to test various adaption measures, for example higher *v* values to simulate a fan or better ventilation, *T*_mrt_ = *T*_i_ for shading, or smaller values of clothing in the PET calculations. The ANNs used for generating the workplace-specific climate projections could possibly be improved by collecting more training data, tuning properties of the ANN structure, using a k-fold cross validation instead of the hold-out method, and/or add additional or optimize the combination of ANN input variables.

EURO-CORDEX data from the models used in this study were not available in a higher temporal resolution than 3 h (Copernicus Climate Change Service, Climate Data Store 2019). Although the requirements for a comprehensive bioclimatic analysis in terms of human biometeorological are generally fulfilled by using 3-h data (Matzarakis 2021), a higher temporal resolution could improve the simulation of extremes and one could quantify mitigation effects of shifting work hours.

Despite the inability to specifically assess different heat mitigation strategies, the presented workplace-specific climate projections can help to assess expected changes at workplaces and identify workplaces that will be most severely affected by climate change and therefore support the selection of effective adaptation measures, as decisions based on current or historical thermal comfort data may not be representative for future conditions (Cheung and Hart 2014). Active cooling systems can be used to reduce indoor thermal stress and health risks for humans, but they are highly energy inefficient and can contribute to excess heat released outdoors, amplifying the nocturnal urban heat island in cities (Salamanca et al. 2014) and generally through their energy consumption cause additional greenhouse gas emissions (Costello et al. 2009).

## Conclusions

We developed a data-driven approach to enable workplace-specific climate projections of *T*_i_ and PET_i_. The approach is based on a combination of indoor sensors, ANN models, and climate projection data. Human thermal comfort and *T*_i_ measured by low-cost sensors at 90 different workplaces were used as training data sets for ANN models predicting indoor conditions as a function of current and past outdoor weather based on the approach of Sulzer et al. (2023). The workplace-specific climate projections were modeled based on simulations of 3-hourly conditions for the future time period 2070–2099. The measurements of the indoor sensors combined with the workplace-specific climate projections can help to identify workplaces where heat stress already is an issue or will be a problem in the future. The use of the workplace-specific ANN models allows estimations about the climate over longer reference periods in the past and in the future, when no measured data are available. Different RCPs can be taken into account in the calculations.

Unsurprisingly, the mean projected increase of *T*_i_ is higher during summer than in winter, due to the impact of heating systems on *T*_i_ in the cold season. The increase of *T*_a_ outdoors is higher than the increase of *T*_i_ at all workplaces for the entire projected future period 2070–2099 as well as during the projected winter and summer months in the future period. Nevertheless, at almost all workplaces, *T*_i_ and PET_i_ can be expected to rise in the projected future in the non-heating season. Further, the frequency of heat stress is higher at most workplaces than outdoors for the historical period as well as for the projected future. Similar to heat events outdoors, it has been shown that heat stress indoors will be more intense, more frequent, and longer lasting in the future for almost all workplace-specific climate projections. The extent of the heat stress increase varies strongly between the different indoor workplaces, implying that room-specific climate projections are needed to assess heat exposure indoors. The information gained by this approach can be useful to identify workplaces where mitigation measures are most urgently needed or will be needed in the future.

## Data Availability

Metadata on the workplaces, indoor data used for training and testing of the ANN models, and the modeled data of the historical (1970–1990) and future time periods (2070–2099) used in this article can be found at 10.5281/zenodo.8229253.
